# Noninvasive Phenotyping of Plant–Pathogen Interaction: Consecutive *In Situ* Imaging of Fluorescing *Pseudomonas syringae*, Plant Phenolic Fluorescence, and Chlorophyll Fluorescence in *Arabidopsis* Leaves

**DOI:** 10.3389/fpls.2019.01239

**Published:** 2019-10-15

**Authors:** Sabrina Hupp, Maaria Rosenkranz, Katharina Bonfig, Chandana Pandey, Thomas Roitsch

**Affiliations:** ^1^Department of Pharmaceutical Biology, University of Würzburg, Würzburg, Germany; ^2^Department of Environmental Sciences, Institute of Biochemical Plant Pathology, Research Unit Environmental Simulation, Helmholtz Zentrum Muenchen, Neuherberg, Germany; ^3^Department of Plant and Environmental Sciences, Section of Crop Science, University of Copenhagen, Copenhagen, Denmark; ^4^Department of Adaptive Biotechnologies, Global Change Research Institute, CAS, Brno, Czechia

**Keywords:** green fluorescence protein (GFP), plant–pathogen interaction, imaging PAM, chlorophyll fluorescence imaging, phenolic compounds

## Abstract

Plant–pathogen interactions have been widely studied, but mostly from the site of the plant secondary defense. Less is known about the effects of pathogen infection on plant primary metabolism. The possibility to transform a fluorescing protein into prokaryotes is a promising phenotyping tool to follow a bacterial infection in plants in a noninvasive manner. In the present study, virulent and avirulent *Pseudomonas syringae* strains were transformed with green fluorescent protein (GFP) to follow the spread of bacteria *in vivo* by imaging Pulse-Amplitude-Modulation (PAM) fluorescence and conventional binocular microscopy. The combination of various wavelengths and filters allowed simultaneous detection of GFP-transformed bacteria, PAM chlorophyll fluorescence, and phenolic fluorescence from pathogen-infected plant leaves. The results show that fluorescence imaging allows spatiotemporal monitoring of pathogen spread as well as phenolic and chlorophyll fluorescence *in situ*, thus providing a novel means to study complex plant–pathogen interactions and relate the responses of primary and secondary metabolism to pathogen spread and multiplication. The study establishes a deeper understanding of imaging data and their implementation into disease screening.

## Introduction

Plants are regularly attacked by several pathogens, such as bacteria, fungi, viruses, oomycetes, nematodes, and others. Due to a lowered performance of an infected plant, pathogen invasion can lead into severe economical losses on economically important field and forest sites (Gutierrez-Arellano and Mulligan, 2018). To defend themselves, plants have developed various strategies in which not only secondary but also carbohydrate metabolism plays complex roles (Trouvelot et al., 2014; Jammer et al., 2015).

Several studies exist on the importance of secondary metabolites in plant defense against pathogen-induced biotic stresses (Großkinsky et al., 2016a; Großkinsky et al., 2016b; Jing et al., 2018; Rosa et al., 2018; Zaynab et al., 2018). Phenolic compounds, the most ubiquitous secondary metabolites in plants, are stress induced and serve in specific roles of plant defense, e.g., as deterrents to pathogens and herbivores and by protecting against UV radiation and oxidative stress. Most of them (flavonoids, tannins, hydroxycinnamate esters, and lignin) have common origin from shikimate acid pathway *via* phenylpropanoids (Ge et al., 2018; Thakur et al., 2018).

The roles of primary metabolites in plant defense against pathogens are less exploited than those of secondary metabolites, even if many biologists have shown a correlation between sugar quantities and plant defense responses (Bonfig et al., 2010; Carvalho et al., 2019; Dong and Beckles, 2019). Furthermore, several studies have proven decrease in photosynthesis simultaneously with pathogen spread on a plant leaf (Bonfig et al., 2006; Berger et al., 2007; Dong et al., 2016; Lu and Yao, 2018; Tischler et al., 2018; Mahlein et al., 2019). The observation may be caused by decreased performance of photosynthetic apparatus and change of the pathogen-attacked site from source to a sink. Alternatively, up-regulation of invertases (Kuska et al., 2018; Su et al., 2018) may lead to observed phenomenon. The physiological background of decreased photosynthetic performance of a pathogen-attacked leaf is, however, still under debate.

Not only plants but also pathogens have developed various strategies to ensure more beneficial outcome in plant–pathogen interactions. Plant pathogens can successfully use plant carbohydrates for their own energy use (Kuska et al., 2018). They are likely to redirect the carbohydrate metabolism in plant leaves to ensure enough nutrients for themselves (Ökmen and Doehlemann, 2014). Several pathogenic species possess sucrose-hydrolyzing enzymes (Chaliha et al., 2018; Kanwar and Jha, 2018), which can help them to secure the plant-provided nutrients directly at the infection site. Indeed, Berger et al. (2007) suggested that change of an attacked leaf from a source to a sink could be due to a pathogen manipulation of plant primary metabolism. Recently, it was shown that certain pathogen species are indeed able to modify the host’s photosynthesis to stay active, thereby creating conditions favorable to its own survival (Xue et al., 2018). Whether the plant-provided nutrients could also enhance the survival of the pathogen in other manners, e.g., by playing a role in pathogenesis, has to be elucidated in the future. Multicolor fluorescence imaging (MCFI) has also been used as a promising tool for disease detection in plant phenotyping (Murchie and Lawson, 2013; Barón et al., 2016; Pérez-Bueno et al., 2016). However, for the detection of plant stress phenotyping, the most commonly applied sensor and imaging techniques are digital RGB (red–green–blue) imaging; spectroscopy; thermography; fluorescence; three-dimensional, by, for example, stereo cameras and LIDAR (light detection and ranging); real-time camera set-ups; RNA-seq analysis; and, to a lesser extent, tomography (Quemada et al., 2014; Großkinsky et al., 2017; Ghosal et al., 2018; Pineda et al., 2018; Dobos et al., 2019; Polonio et al., 2019; Sperschneider, 2019).

Accumulation of bacteria in plants was studied mainly by reisolation of the bacterial cells (Raacke et al., 2006; Aydi-Ben-Abdallah et al., 2019; Liu et al., 2019). Similarly, the analysis of phenolic compounds in plant tissues commonly proceeded through diverse extraction methods (Torti et al., 1995; Giorgetti et al., 2018; Proestos et al., 2018). The techniques provide an invasive method to achieve information on pathogen accumulation or plant defense response. However, the disadvantages of invasive techniques are clear; the determination has to be done from detached leaves; the changes cannot be followed over time; and moreover, the precise location of the pathogen is not possible.

To assess the impact of biotic stress on host plant, various imaging techniques are currently used in plant physiology (Barón et al., 2016). Some of these techniques include MCFI and chlorophyll fluorescence (Chl-F) imaging. Plant health status is monitored by MCFI, and it is based on recording the blue (F440), green (F520), red (F680), and far red (F740) fluorescence by leaves when they are excited with UV light (Polonio et al., 2019). Particularly, the blue–green fluorescence is a valuable technique to study secondary metabolism, because phenolic compounds from the phenylpropanoid pathway are the primary emitters of that fluorescence. The extent of absorbance of light by the epidermal polyphenols can be derived on the basis of the ratio of Chl-F emission intensities induced by a standard red beam and a Ultraviolet-visible spectroscopy (UV-VIS) beam. Similarly, red Chl-F emitted by photosystem II (PSII) provides information on the photosynthetic performance of plants in terms of activity and indirect information on the CO_2_ assimilation rate (Murchie and Lawson, 2013).

Novel technologies such as hyperspectral imaging and Chl-F imaging offer an elegant, noninvasive means to explore indirectly the bacteria spread within the plant tissue (Rolfe and Scholes, 2010; Großkinsky et al., 2017; West et al., 2017; Bohnenkamp et al., 2019; Kuska et al., 2019). In our own study (Bonfig et al., 2006), the decrease in maximum PSII quantum yield after avirulent *Pseudomonas syringae* infection was detectable already 3 h after the inoculation of bacteria into the tissue. However, the method is not directly measuring the accumulation of bacteria, and the changes in photosynthesis could be due to other, undetermined, reasons. A direct noninvasive manner to locate and quantify bacterial pathogen in the plant leaf tissue was missing until Wang et al. (2007) decided to transform *P. syringae* cells with a plasmid containing bright fluorescing green fluorescence protein (GFP) uv-gene. The group was able to monitor the bacterial expansion in the whole plant level under long-wavelength UV light. Later, confocal laser scanning microscopy (Riedel et al., 2009) and fluorescence microscopy (Parente et al., 2008) were applied to detect GFP-expressing bacteria. Labeling prokaryotic cells by GFP has become a routinely applied technique to visualize cells in plant living tissue (Lozoya-Pérez et al., 2018; Yang et al., 2019).

Our interest is, in addition to detecting bacterial cells, to follow a pathogen invasion on a leaf by simultaneously monitoring direct changes in plant primary and secondary metabolisms. Here we present a technique to monitor different fluorescing sources on plant leaves *in situ* by using imaging PAM fluorescence (Heinz Walz GmBH) system as a tool. In the present study, we detect the GFP, Chl-F, and phenolic fluorescence within a short time from one intact leaf by utilizing different wavelengths and filters. The possibility to combine the three measurements in one instrument provides a clear advantage in characterization of the plant–pathogen interactions in the future. For initializing and valuing the technique, *Arabidopsis thaliana* and *P. syringae* were used as model systems. *Pseudomonas syringae* is a hemibiotrophic pathogen (Preston, 2000) that can invade several, also economically important plant species.

## Materials and Methods

### Plant and Bacterial Materials

*Arabidopsis thaliana*, cv. Columbia 0, were cultivated at 22°C, L9:D15, and a photosynthetic photon flux density of 180 μmol photons m^−2^ s^−1^ in climate chambers (Binder, Germany). We used 5- to 8-week-old *Arabidopsis* rosettes in the experiments. One of the first fully expanded leaves was chosen for the measurements. One *Arabidopsis* plant was treated as one biological replicate.

*Pseudomonas syringae* DC3000 and DC3000rpm were cultured in 28°C either on LB agar plates or by shaking in Kings medium B. The medium contained the appropriate antibiotics as follows: 50 µg mL^−1^ rifampicillin for both *P. syringae* strains, 5 µg ml^−1^ tetracyclin for the avirulent strain, and 100 µg ml^−1^ kanamycin for pPNptGreen-expressing bacteria. For plant infection, *P. syringae* were harvested by centrifuging and resuspended in 10 mM MgCl_2_ until optical density (OD)_600_ = 0.2, which is equal to approximately 1 × 10^8^ cell-forming units (cfu) ml^−1^. Plants were infected by infiltrating the appropriate bacterial suspension by 1-ml plastic syringe (without needle) through the stomata into the leaf tissue. The individuals that were treated with the pathogens were selected randomly among the cultivated plants.

Electrocompetent *P. syringae* cells were obtained by growing bacteria in 500 ml of Kings medium B under optimal conditions until OD_600_ = 0.6 ± 0.1. The bacteria were harvested by centrifuging and resuspended to 500 ml 10% glycerol (4°C). The harvest was repeated, and after each harvest, the pellet was resuspended first to 250 ml and then to 150 ml and finally to 3 ml cold 10% glycerol. The obtained bacteria cells were stored at −80°C until use.

### Creating pPNptGreen Construct and Transforming it Into *P. Syringae*

The plasmid pPNptGreen (13,199 bp) carrying a GFP gene sequence and kanamycin resistance was obtained as a gift from G. Beattie (Department of Plant Pathology, Iowa State University, Iowa, USA). The competent *P. syringae* cells were transformed with pPNptGreen by electroporation (2.5 kV) and spread on LB agar plates. The colonies carrying the pPNtpGreen construct were selected by kanamycin resistance and additionally by detecting green fluorescence under UV light.

### Measurement of the Growth of the Fluorescing and Wild-Type Bacteria in *Arabidopsis*

The reisolation of bacterial cells from plant leaves (Raacke et al., 2006) was applied to compare the growth of fluorescing bacteria to that of the wild type. Four leaves per plant were infected with 10^5^ cfu ml^−1^ of *P. syringae*. The infection sites were harvested with a corkbore (*r* = 0.7 cm) 24, 48, and 72 h after the infiltration. All the four infection sites from one plant were pooled together and fine powdered in 500 µL 10 mM MgCl_2_, with a pestle in an Eppendorf tube. The obtained suspension was diluted with MgCl_2_ until 1 ml and further until 1:100 or up to 1:100,000. A hundred microliters of each dilution was spread on agar plates; the plates were grown under 28°C for 48 h after which the colonies were calculated.

### Quantifying the Fluorescence Signal From *P. Syringae*

Known concentrations (10^10^, 10^9^, 10^8^, 10^7^, 10^6^m, and 10^5^ cfu ml^−1^) of fluorescing *P. syringae* in 10 mM MgCl_2_ were used to valuate two different methods:

First, the fluorescence was quantified by a fluorometer (Fluoroscan, Ascent, Germany). The *P. syringae* suspensions (200 µL) in different concentrations were pipetted on a black microtiter plate. The GFP was excited at 485 nm, and fluorescence was measured at 538 nm. The background fluorescence of nonfluorescing control bacteria was subtracted from the obtained values. Second, to validate the signal detected by imaging PAM, the fluorescence of single *P. syringae* drops in different concentrations on black, nonfluorescing background was quantified. Photographs were taken with imaging PAM (for details of the technique, see below), and the fluorescence signal in the middle of the drop was measured. As a control, the nonfluorescing *P. syringae* strains were used.

### Detecting GFP Under Fluorescence Binocular

The fluorescing bacteria were detected with fluorescence binocular, which was equipped with a special GFP3 filter (instruction manual imaging PAM, Heinz-Walz GmbH, Germany). The fluorescence were measured each 24 h until 96 h after the infection. Photographs of the fluorescing bacteria were taken with a camera (Spotlight Color) and analyzed by the photograph software SpotAdvanced.

### Detection of Chl-F and GFP by Imaging PAM

Chlorophyll fluorescence measurements were performed as described in Bonfig et al. (2006). A maximum saturation pulse was applied on dark adapted (ca. 20 minutes) plants. Further fluorescence parameters were subsequently measured with actinic light intensity set at 76 µmol m^−2^ s^−1^. Measurements were performed every 20 s, from 50 to 290 s, duration of the light pulses being set at 8 (instruction manual imaging PAM, Heinz-Walz GmbH). We report PSII quantum yield (Y(II) = (Fm′ − F′)/Fm′) and Fv/Fm (Fv/Fm = (Fm − Fo)/Fm) similar as has been previously described (for review e.g. Maxwell and Johnson, 2000; Murchie and Lawson, 2013). In addition to Chl-F, GFP was detected by imaging PAM (Walz, Germany) equipped with a special long-pass filter with an angle of 645 nm. The GFP was excitated at 450 nm. The measurements were performed with maximum intensity of measuring light and gain (the amplitude of fluorescence signal) = 8 (instruction manual imaging PAM, Heinz-Walz GmbH). Green fluorescent protein fluorescence was detected from the below sites of the leaves.

### Detection of Phenolic Fluorescence by Reactive Oxygen Species Head

Phenolic compounds were detected by a special application developed for imaging PAM. The standard blue power LED lights were substituted with special UV-A power LEDs having an emission peak at 365 nm. All the wavelengths above 400 nm were filtered from the excitation light and a short-pass interference ﬁlter blocked the transmission above 650 nm, which is essential for excluding chlorophyll ﬂuorescence from the detected signal. The reactive oxygen species (ROS) head was operated analog to imaging PAM, as described previously by Hideg and Schreiber (2007). Measuring light is applied as short (10–200 ls) pulses at low frequency (1–8 Hz). Two images are measured: one during the pulse and one directly afterward, from which a difference image was derived. This eliminates eventually disturbing ambient background light (Hideg and Schreiber, 2007). Phenolic fluorescence was detected from the below sites of the leaves.

### Statistical Analysis

Statistical and correlation analyses were performed with SPSS for Windows (release 15.0) and Sigma Plot for Windows (version 10.0). Linear regression analysis was performed to describe the dependency of fluorescence intensity on bacterial density.

## Results and Discussion

### Selection of Transgenics

The possibility to transform a fluorescing protein into a prokaryote is a promising tool to follow bacterial infection in a nondestructive manner in plants (Wang et al., 2007; Parente et al., 2008). In the present work, GFP was successfully transformed into two strains of bacterial pathogen *P. syringae*; in a virulent strain pv. *tomato* DC3000 and in an avirulent strain pv. *tomato* DC3000rpm. The positive transformants were selected by kanamycin resistance and additionally by picking the transgenic, green-fluorescing colonies from agar plates under UV light. The highest fluorescing colonies were chosen for further studies.

### Detection of GFP-Fluorescing Bacteria in Plant Leaves by Binocular

**Figure 1:**
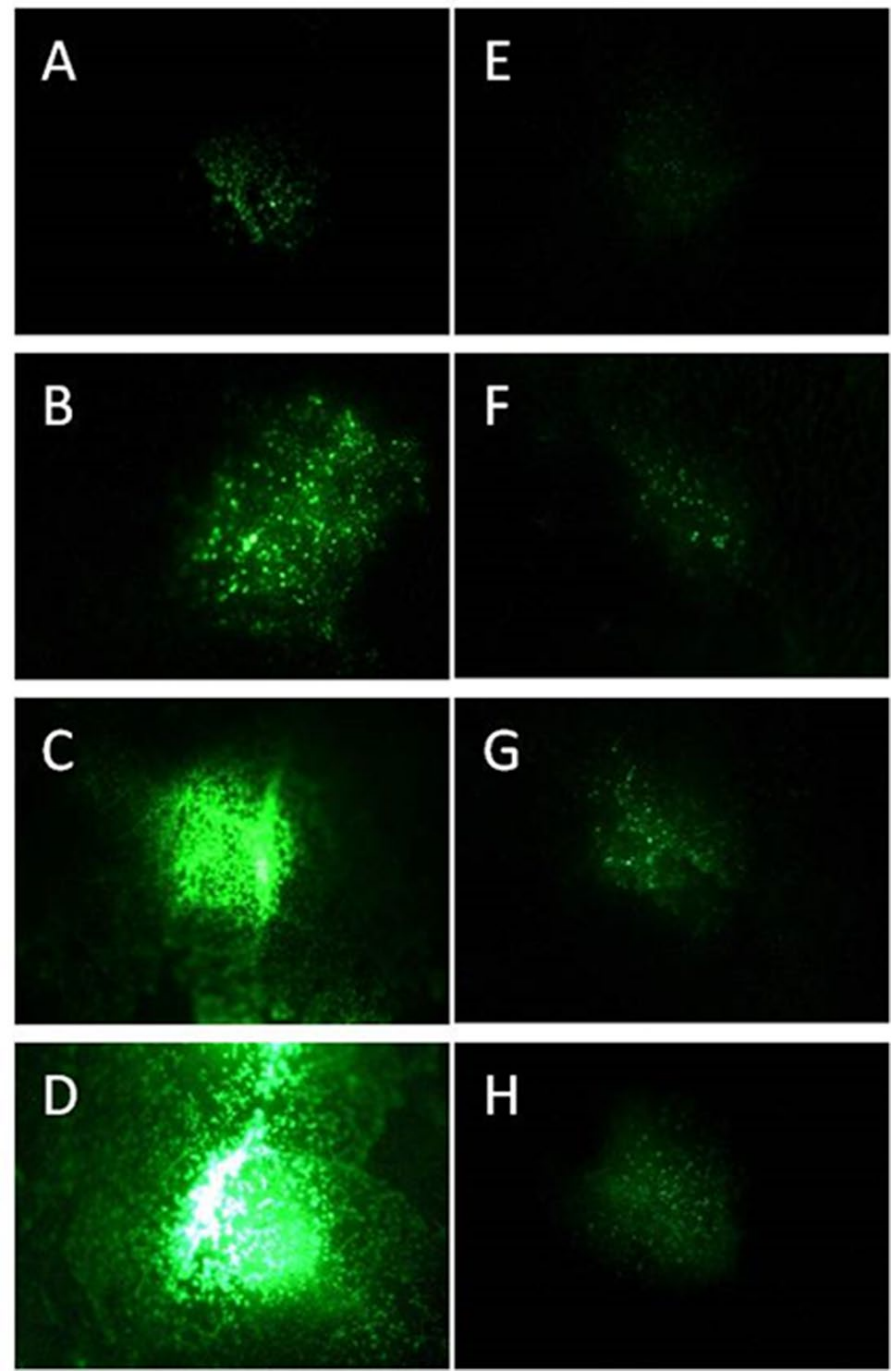
Progeny of GFP synthesizing *P. syringae* DC3000 **(A**–**D)** and DC3000rpm **(E**–**H)** detected as fluorescence signal 24 h **(A**, **E)**, 48 h **(B**, **F)**, 72 h **(C**, **G)**, and 96 h **(D**, **H)** after infiltration with 1 × 10^7^ cfu ml^−1^ for DC3000gfp and 1 × 10^8^ cfu ml^−1^ for DC3000rpm_gfp.

The GFP-transformed *P. syringae* DC3000 were detected in *Arabidopsis* leaves 24 h after an infection with 1 × 10^7^ cfu ml^−1^ pathogen (**Figure 1A**) under a binocular. When lower concentration (≤1× 10^6^ cfu ml^−1^) of pathogens was initially applied or dip-inoculation-technique used, the fluorescence was detected earliest at 48 h after the infection. An application of 1 × 10^5^ cfu ml^−1^ was detectable only 96 h after the infection (data not shown).

The progeny of both *P. syringae* DC3000 strains (each applied in concentration of 1 × 10^7^ cfu ml^−1^ on individual leaves) was followed by binocular over 96 h. At the first time point (24 h after the infection), the virulent strain was hardly detectable; however, the fluorescence intensity increased by each 24-h period and was at its strongest 96 h after the infection (**Figures 1A–D**). Unlike the virulent strain, the fluorescent signal of avirulent *P. syringae* DC3000rpm strain was not well detectable. First, the detection was possible only when at least 1 × 10^8^ cfu ml^−1^ bacterial concentration was initially infiltrated into the leaf tissue. Twenty-four hours after the infection, only a very low signal was detected (**Figure 1E**). The signal increased slightly over the time, and the signal was stronger in later time points, when the time points 24 h and 72 h or 48 h and 96 h were compared to each other (**Figures 1E–H**). The fact that avirulent strain of *P. syringae* showed lower increase in fluorescence signal over time than the virulent strain was likely due to an incompatible interaction between avirulent strain and the plant. The fast reaction to avirulent strain by programmed cell death testifies for incompatible interaction (Bonfig et al., 2006) and for faster defense response against avirulent than virulent strain in *Arabidopsis*. The fast defense response against the microbial pathogen was followed by low pathogen progeny and, logically, low GFP fluorescence that partly remained under the detection limit. Due to these complications, the avirulent strain was excluded from part of the further studies.

**Figure 2:**
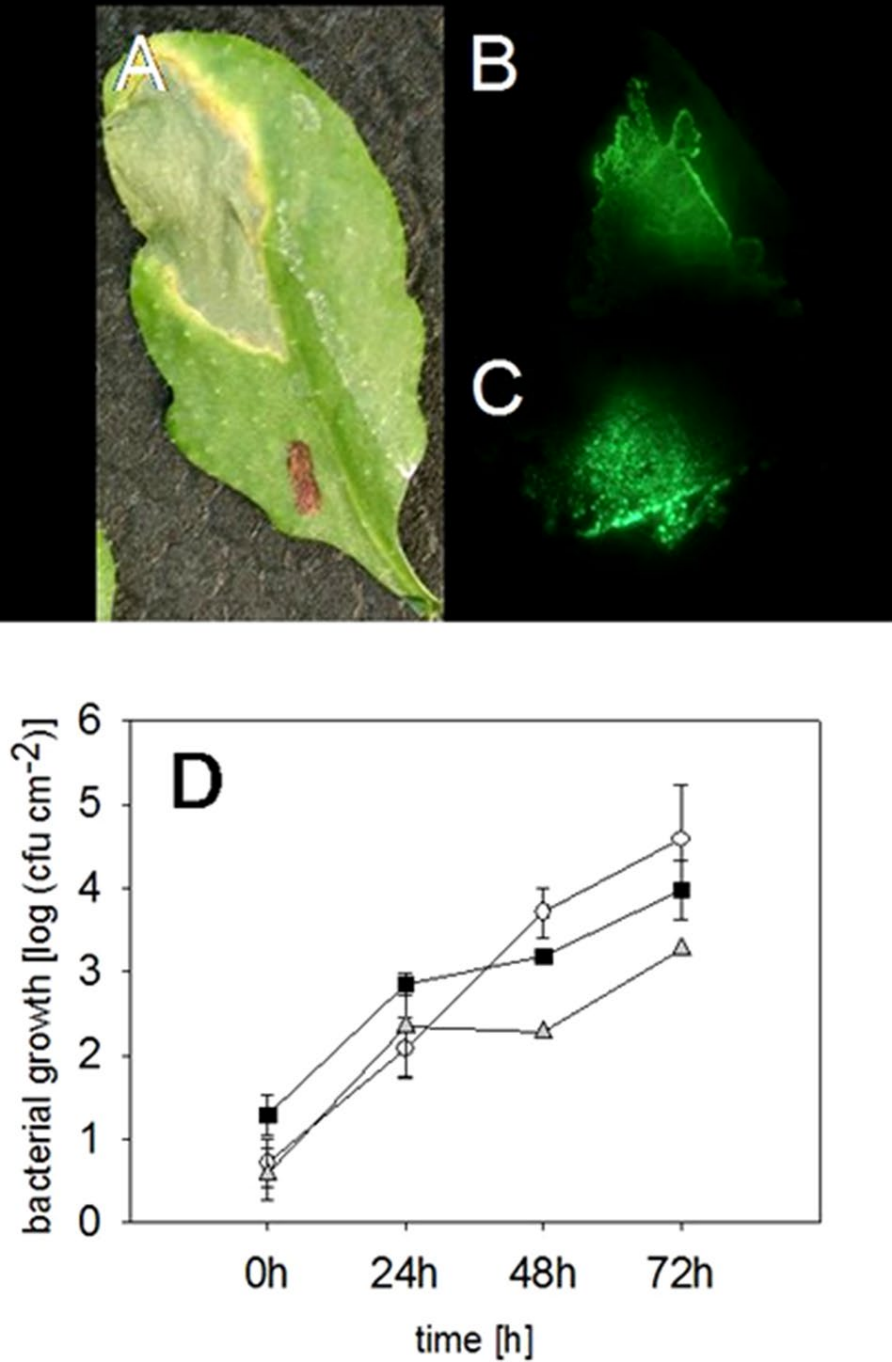
Locating and quantifying the *P. syringae* DC3000_gfp cells in differentially infected *Arabidopsis* leaves. Typical symptoms in the *Arabidopsis* leaves due to an infection with *P. syringae* applied by a needless syringe **(A)** and the location of the bacterial cells in the same leaf by a fluorescence signal 2 weeks after the infection **(B)**. The highest fluorescing signal was detected in the leaf veins compared to other tissue 72 h after an infection with *P. syringae* **(C)**. The accumulation of fluorescing and nonfluorescing *P. syringae* DC3000 and fluorescing DC3000rpm cells in *Arabidopsis* leaves. DC3000 (▪); DC3000gfp (○); DC3000rpm_gfp (▲); n = 3 **(D)**. No significant differences (Kruskal-Wallis) were found between the different strains.

As expected, an infected leaf showed further developed necrosis in the middle of the infiltration site than on the edges of it (**Figure 2A**). Interestingly, 2 weeks after the infection with the virulent *P. syringae* strain, the highest fluorescence signal was measured on the edges of the infection site, whereas the signal was lower directly in the middle of the infiltration site (**Figure 2B**). Thus, the strongest fluorescence signal was detected on the areas in which the bacteria invaded so far untouched plant cells. Furthermore, our data show that the bacterial density was at its highest on the edges of the infection site 2 weeks after the infection, whereas a lower bacterial density at the necrotic sites was found. The result suggests that the bacteria spread on the plant leaf from necrotic spot further into not yet infected areas.

Furthermore, in the infected *Arabidopsis* leaves, it was obvious that the virulent *P. syringae* cells accumulated stronger in the vascular tissue of the plant leaves than on the other sites. Higher fluorescence signal was often, even if not always, found in the middle vein or in the smaller veins of the plant leaves (**Figure 2C**). Such an accumulation was detected at different time points by binocular, but only when at least 1 × 10^8^ cfu ml^−1^ bacterial concentration was initially applied into the leaves.

Whether virulent *P. syringae* use the veins to move from one site to another or prefer them due to a higher nutrient quantity is not known at present.

### The Progeny of Wild-Type and GFP-Transformed *P. Syringae*

**Figure 3:**
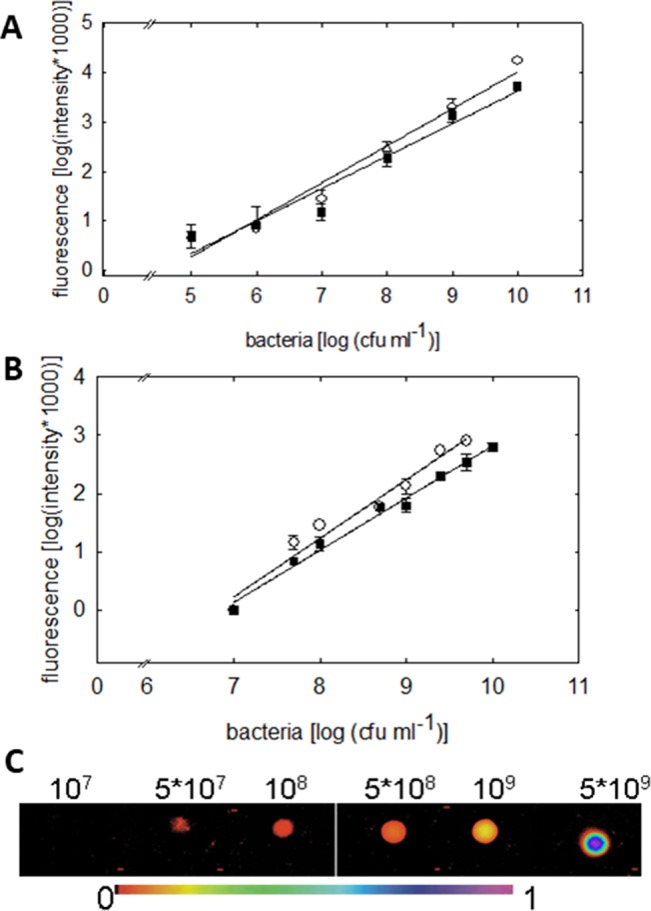
Quantification of the green fluorescence intensity emitted by *P. syringae* DC3000_gfp (○) or DC3000rpm_gfp (▪) cells. **(A)** Fluorometric quantification of cells in six different concentrations, from 1 × 10^5^ up to 1 × 10^10^ cfu ml^−1^, is shown. The fluorescence signal shows linear dependency on the concentration of the bacteria (*R* = 0.982; *y* = 0.748*x* − 3.468 (DC3000) and *R* = 0.9750; *y* = 0.656*x* − 2.937 (DC3000rpm); n = 4 ± 2). **(B)** Quantification of green fluorescence intensity by imaging PAM. The fluorescence was measured by imaging PAM from 20 µL of *P. syringae* cells in 10 mM MgCl_2_ in increasing concentrations. The fluorescence signal shows linear dependency on bacterial concentration; *R* = 0.98; *y* = 1*x* − 6.8 (DC3000) and *R* = 0.99 = 0.99; *y* = 0.89*x* − 6.12 (DC3000rpm); n = 4 ± 1. In **(C)**, an example of fluorescence signals in various *P. syringae* concentrations is shown (from 1 × 10^7^ up to 5 × 10^9^ cfu ml^−1^) detected by imaging PAM. The false scale color is given in the bottom.

The fluorescing *P. syringae* developed symptoms in a similar manner with the nonmarked wild-type strain. Infiltration of the virulent, fluorescing strain into leaf tissue developed necrosis surrounded by so-called “chlorotic halos” at the infection site (**Figure 2A**). We also found no differences in the accumulation of the fluorescing and wild-type pathogens (for the time point 72 h: Kruskal-Wallis, *P* = 0.191, n = 3) (**Figure 3A**). The bacteria reached a concentration of approximately 615 ± 230 cfu cm^−2^ 24 h after the infiltration and the amount of cfu was approximately doubled within each further 24-h periods. Seventy-two hours post infection, a concentration of approximately 4.5 × 10^4^ cfu ml^−1^ was reached (**Figure 2D**).

As the fluorescing bacterial cells grew similar to the nonmarked cells, the plasmid pPNtpGreen likely did not interfere the plant–pathogen interaction. Wang and colleagues, who studied several different *P. syringae* strains (Wang et al., 2007), previously showed similar results. In general, our data prove that the fluorescing bacteria can be used instead of nonfluorescing wild-type bacteria in wide range of studies that aim to explore *P. syringae* interaction with its host.

### Quantification of GFP-Transformed *P. syringae*

Fluorometric assay, compared to the traditional reisolation technique of bacteria, provides an easy and accurate tool to rapidly quantify the bacterial density on a plant leaf. The bacterial densities were detectable down to 1 × 10^6^ cfu ml^−1^ by the fluorometric assay. The fluorescence signal detected by fluorometer showed linear increase with increasing concentration of cell units (*R* = 0.982 for virulent and *R* = 0.975 for avirulent strain, **Figure 3A**). No differences in the signal between virulent and avirulent bacteria were found (Mann-Whitney *U*, *P* > 0.1 for all the time points, n = 3 ± 1), testifying for a comparable expression of the GFP in both of the strains.

We moreover quantified the fluorescence signal of different *P. syringae* concentrations by imaging PAM. Measuring the fluorescence of *P. syringae* drops in different concentrations (**Figure 3C**) made it possible to validate the GFP detection by imaging PAM (**Figure 3B**). The lower detection limit of imaging PAM was identified at 5 × 10^7^ cfu ml^−1^, and the saturation of the signal was detected at 1 × 10^10^ cfu ml^−1^. The fluorescence signal depended linearly on bacterial concentration, *R* = 0.98 for virulent and *R* = 0.99 for avirulent strain. In support with the fluorometric quantification, also here no differences between virulent and avirulent strain were found (Mann-Whitney *U*, *P* > 0.1 for all the time points, n = 4 ± 1), further verifying a comparable expression of the GFP in both of the strains. Detection of pathogen in plants on multiple scales facilitates the advancement and current development.

### Simultaneous Detection of GFP and Chl-F by Imaging PAM

**Figure 4:**
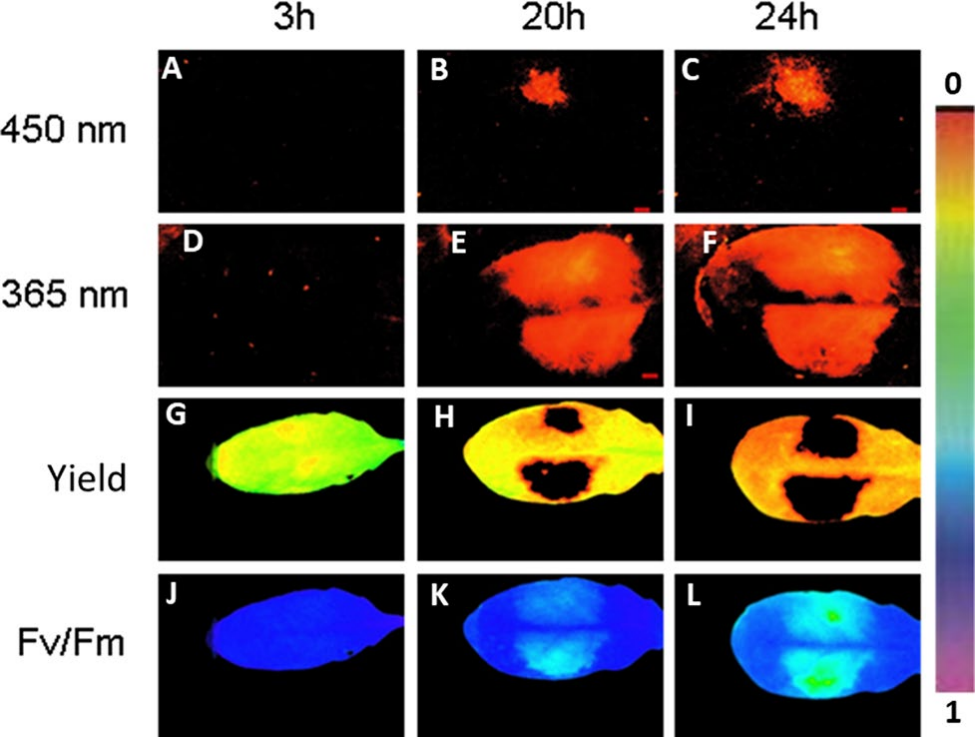
An example of *Arabidopsis* leaf infected with GFP-labeled *P. syringae* DC3000 on one side of the leaf (upper side) and with wild-type *P. syringae* on the other site of the leaf (lower side), both in concentration 1 × 10^7^ cfu ml^−1^. The fluorescence signal is detected after 3, 20, and 24 h by exciting with either 450 nm (imaging PAM) **(A**–**C)** or 365 nm (ROS head) **(D**–**F)**. Quantum efficiency of PSII **(G**–**I)** and maximum efficiency of PSII (Fv/Fm) **(J**–**L)** are shown for the same time points. The false color scale is given on the right.

Next we followed simultaneously the progeny of fluorescing virulent *P. syringae* in *Arabidopsis* leaves and the plant primary metabolism performance in a noninvasive manner by imaging PAM. Infection with GFP-marked *P. syringae* was detectable by imaging PAM (excitation with 450-nm wavelength) 20 and 24 h post infection at which time points fluorescence from wild-type bacteria site was not yet detectable (**Figures 4A–F**).

Imaging PAM made it possible to visually prove that the bacterial cells were indeed located on the area in which also plant’s photosynthetic performance was affected. On the same leaf site on which bacteria were inoculated, the photosynthetic performance of the plant leaves decreased in synchrony with the spreading bacteria. A decrease in quantum yield of PSII (**Figures 4G–I**) and in maximum efficiency of PSII (**Figures 4J–L**) was detected from the initial values. The result supports the previous studies showing that reducing photosynthesis is an effective method to defend against biotrophic pathogens (Berger et al., 2007; Garavaglia et al., 2010). No differences were found in photosynthetic performance between the GFP-labeled and wild-type *P. syringae–*infected leaves. The lowest maximum quantum yields of PSII (Fv/Fm) were detected at the very sites where the bacteria were initially inoculated (**Figure 4**).

The detection of the fluorescing, avirulent *P. syringae* strain was possible only ≥24 h post infection and only when initially a high amount of *P. syringae* (1 × 10^8^ cfu ml^−1^) was applied (data not shown). At these later time points (≥24 h), it was not possible to distinguish GFP from plant phenolic fluorescence by imaging PAM.

The visualization of the bacterial cells by imaging PAM can be especially useful in investigating the role of plant primary metabolites in plant defense responses. The location of the bacteria can also be assigned more accurately than by, e.g., reisolation of bacteria. One of the advantages of GFP detection by imaging PAM over binocular is the possibility to take all the fluorescence pictures in similar position with exactly the same distance between the leaf and camera.

### Combining GFP, Phenolic, and Chl-F Detections *In Situ*

The fluorescing nature of the phenolic compounds allows these secondary metabolites to be detected under UV or blue light. As GFP can be detected with the excitation peak at 450 nm, at which wavelength also phenolic fluorescence is excited, we applied furthermore a special application of imaging PAM, so-called ROS head (Hideg and Schreiber, 2007), which sends wavelength of 365 nm. Using this special application, we detected phenolic fluorescence, in *Arabidopsis* leaves infected with GFP synthesizing *P. syringae* DC3000 on one leaf side and with wild-type *P. syringae* on the other side. Our results reveal that, contrary to imaging PAM, by ROS head a strong plant fluorescence signal was detected 20 and 24 h post infection with any *P. syringae* strain (**Figures 4E, F**). Both of the wavelengths (UV in ROS head and blue light in imaging PAM) were thus used in the studies to be able to distinguish plant autofluorescence from GFP.

**Figure 5:**
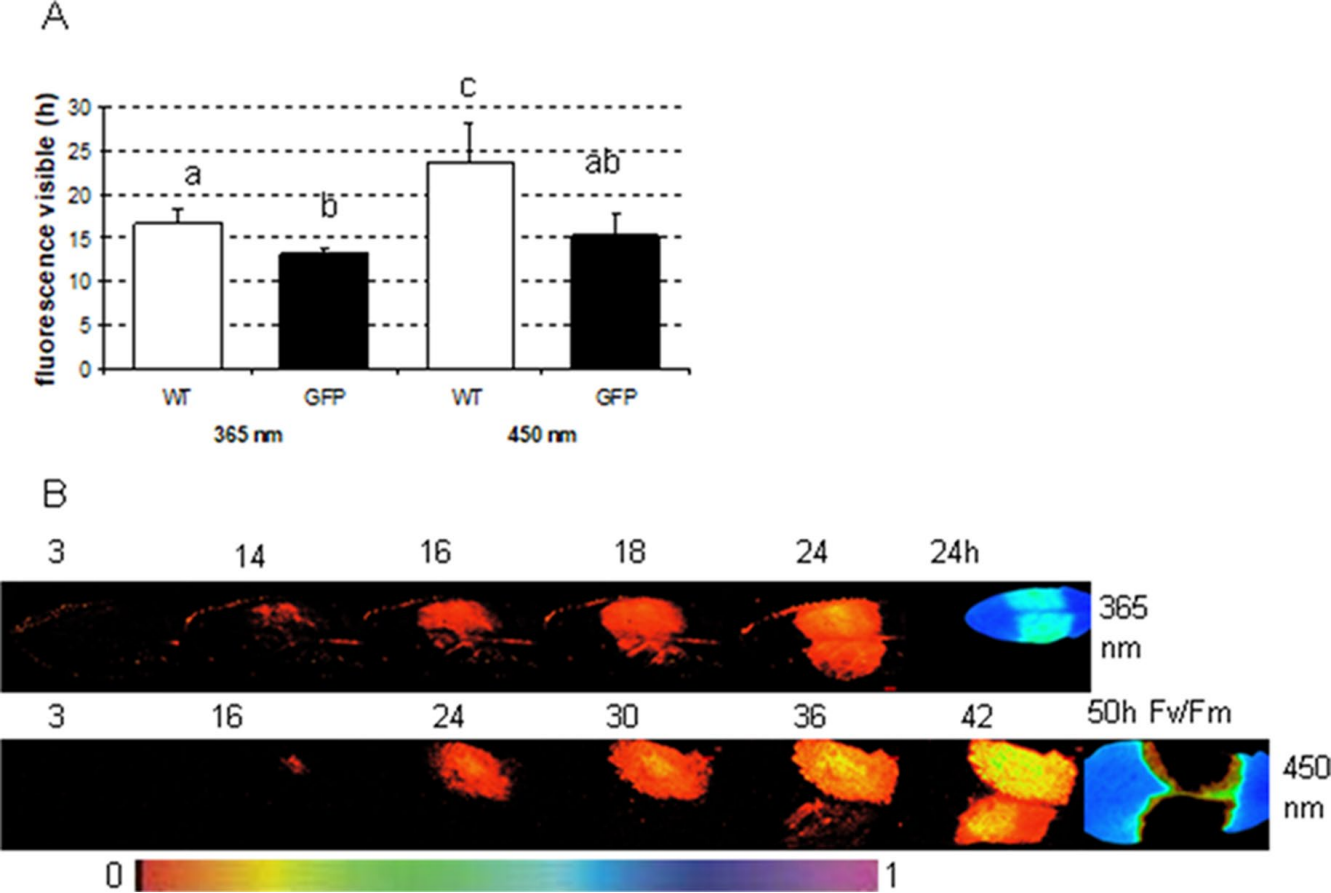
The time in which fluorescence signal is detectable from *Arabidopsis* leaves infected either with wild-type or GFP-labeled *P. syringae* DC3000. **(A)** The fluorescence signal detection limit with either excitation by imaging PAM (450 nm) or by ROS head (365 nm) when *Arabidopsis* leaves were infected with 1 × 10^7^ cfu ml^−1^ type (WT) or GFP-labeled (GFP) *P. syringae* DC3000. Different letters represent the significant differences between different treatments and methods (*P* < 0.01; Student *t* test, n = 6 ± 1). **(B)** An example of the fluorescence signal detection by ROS head (upper row) and imaging PAM (lower row) over time when one side of the *Arabidopsis* leaves was infected with GFP synthesizing (upper site) and the other side with wild-type (lower site) *P. syringae* DC3000. Maximum efficiency of PSII (Fv/Fm) in the end of the experiment is shown for each experiment on the right. False color scale is given in the bottom of the figure.

To determine in which time frames GFP and phenolic fluorescence can be distinguished from each other, the fluorescence signal from several infected plant leaves (one side of the leaf infected with GFP labeled, the other side with nonmarked *P. syringae*) was recorded each half an hour during at least 24 h. The measurements were done by applying either 365 nm (ROS head), so that only plant phenolic fluorescence was excited, or 450 nm (imaging PAM), which excites both GFP and phenolic fluorescence. The results show that with imaging PAM GFP is detectable 15.3 ± 2.5 h after infection, whereas phenolic fluorescence can be seen only 23.7 ± 4.4 h after the infection (**Figures 5A, C**; *P* < 0.01 (Student *t* test).

The recordings with ROS head (excitation at 365 nm) show that phenolic fluorescence appears approximately at the same time point (16.6 ± 1.7 h) (**Figures 5A, B**) as GFP can be detected with imaging PAM. Thus, imaging PAM was “blind” to phenolic fluorescence until a certain level of fluorescence signal was achieved. With ROS head, the GFP-labeled bacteria were detected probably due to combination of phenolic fluorescence and GFP signal already 13 ± 0.7 h post infection. The earlier detection point is probably due to GFP that can be excited by UV light in certain extent. Our results show that with certain limits GFP can be distinguished from phenolic fluorescence and vice versa.

In the present study, phenolic compounds could be easily spatially located without damaging the leaf. In addition to spatial location, the time course of the induction of secondary metabolites after a pathogen attack was followed over several days with very short time intervals. With the help of ROS head, the measurements were recorded automatically over a longer period, which reduced the need of labor. A certain, minimum level of *P. syringae* is necessary before a detection of fluorescing bacterial cells is possible by imaging PAM, which is a disadvantage of the application. Another limitation is that the fluorescence signal is a mixture of GFP and plant phenolic fluorescence signals already approximately 24 h after the infection. Thus, the period in which GFP can be detected at 450 nm without background signals is relatively small, and the appearance of other signals has to be always excluded by nontransformed controls. Interestingly, however, by ROS head, phenolic fluorescence signal can be detected in the same time frame as GFP by imaging PAM. Instead of concentrating only to GFP detection, it might be of interest to use the detection of phenolic compounds to suspect a pathogen attack in several research applications. The initiation of plant defense metabolism could be proven well before physiological changes in the leaves are visible. Most interestingly, detecting phenolic fluorescence does not acquire transgenic bacterial lines.

Taken together, the different wavelengths between phenolic fluorescence, GFP fluorescence, and Chl-F allow the detection of all three from a single leaf within minutes. These imaging techniques enable a novel kind of insight to the plant pathogen interactions and could be applied for diverse research purposes. It is our interest to further optimize the technique to simultaneous image the plant performance and pathogen progeny *in situ*. Should this be possible in microscopic scale, an even more sensitive technique can be developed for visualization of plant–pathogen interactions. Further, this proof-of-concept study needs to be tested, verified, and validated with other pathosystem and expanded to other parameters, which can be determined in a noninvasive way. For initializing and valuing the technique, *A. thaliana* and *P. syringae* are used as model systems. *Pseudomonas syringae* is a hemibiotrophic pathogen that can invade several, also economically important plant species. Recent advancement and current development are facilitating the detection of pathogen in plants on multiple scales. Although it is challenging regarding the diverse type of pathogen, we must explore multiscale approach by possibility to combine this technique with other types of noninvasive analysis either in combination with reporter construct or fluorescent dye–like monitoring pH changes and ROS. Finally, within a truly holistic functional phenomics approach, the image-based, noninvasive phenotyping needs to be complemented by physiological phenotyping (Großkinsky et al., 2017). Thus, the optical signals need to be related to cell and ecophysiological parameters by methods such as the determination of enzyme activity signatures (Jammer et al., 2015) and phytohormone profiles (Großkinsky et al., 2014).

## Data Availability Statement

The raw data supporting the conclusions of this manuscript will be made available by the authors, without undue reservation, to any qualified researcher.

## Author Contributions

SH, MR, and KB conducted the experimental work and analyzed the data. MR drafted a preliminary version of the manuscript, and CP made an updated and revised version of the manuscript and finalized the manuscript for publication. TR designed the project and contributed to the preliminary and final version of the manuscript. All authors discussed the results.

## Funding

The work of TR was supported by the Ministry of Education, Youth and Sports of CR within the National Sustainability Program I (NPU I), grant LO1415.

## Conflict of Interest

The authors declare that the research was conducted in the absence of any commercial or financial relationships that could be construed as a potential conflict of interest.
